# The Effects of Prolonged Treatment with Cemtirestat on Bone Parameters Reflecting Bone Quality in Non-Diabetic and Streptozotocin-Induced Diabetic Rats

**DOI:** 10.3390/ph16040628

**Published:** 2023-04-21

**Authors:** Monika Martiniakova, Veronika Kovacova, Vladimira Mondockova, Karol Svik, Piotr Londzin, Joanna Folwarczna, Marta Soltesova Prnova, Milan Stefek, Radoslav Omelka

**Affiliations:** 1 Department of Zoology and Anthropology, Faculty of Natural Sciences and Informatics, Constantine the Philosopher University in Nitra, 949 01 Nitra, Slovakia; 2 Department of Botany and Genetics, Faculty of Natural Sciences and Informatics, Constantine the Philosopher University in Nitra, 949 01 Nitra, Slovakia; 3 Centre of Experimental Medicine, Institute of Experimental Pharmacology and Toxicology, Slovak Academy of Sciences, 841 04 Bratislava, Slovakia; 4 Department of Pharmacology, Faculty of Pharmaceutical Sciences in Sosnowiec, Medical University of Silesia in Katowice, 41-200 Sosnowiec, Poland; 5 Faculty of Informatics and Information Technologies, Slovak University of Technology in Bratislava, 842 16 Bratislava, Slovakia

**Keywords:** cemtirestat, aldose reductase inhibitor, antioxidant activity, cortical bone, trabecular bone, bone microstructure, bone mechanical properties, STZ-induced diabetic rats, type 1 diabetes mellitus

## Abstract

Cemtirestat, a bifunctional drug acting as an aldose reductase inhibitor with antioxidant ability, is considered a promising candidate for the treatment of diabetic neuropathy. Our study firstly examined the effects of prolonged cemtirestat treatment on bone parameters reflecting bone quality in non-diabetic rats and rats with streptozotocin (STZ)-induced diabetes. Experimental animals were assigned to four groups: non-diabetic rats, non-diabetic rats treated with cemtirestat, diabetic rats, and diabetic rats treated with cemtirestat. Higher levels of plasma glucose, triglycerides, cholesterol, glycated hemoglobin, magnesium, reduced femoral weight and length, bone mineral density and content, parameters characterizing trabecular bone mass and microarchitecture, cortical microarchitecture and geometry, and bone mechanical properties were determined in STZ-induced diabetic versus non-diabetic rats. Treatment with cemtirestat did not affect all aforementioned parameters in non-diabetic animals, suggesting that this drug is safe. In diabetic rats, cemtirestat supplementation reduced plasma triglyceride levels, increased the Haversian canal area and slightly, but insignificantly, improved bone mineral content. Nevertheless, the insufficient effect of cemtirestat treatment on diabetic bone disease does not support its use in the therapy of this complication of type 1 diabetes mellitus.

## 1. Introduction

Diabetes mellitus (DM) represents a current global epidemic and a complex metabolic disorder characterized by chronic hyperglycemia caused by insulin resistance, impaired insulin secretion, or both. The most common classifications include type 1 DM (T1DM) and type 2 DM (T2DM). Whilst T1DM is caused by an absolute lack of insulin on an autoimmune or idiopathic basis, T2DM is characterized by the presence of insulin resistance and relative insulin deficiency [1,2]. Many of the most serious complications of DM are due to oxidative damage, which is induced by hyperglycemia and hyperlipidemia, the defining characteristics of this disease. Generally, diabetic complications can be divided into macrovascular and microvascular [3,4]. Both microvascular and macrovascular complications are a major cause of morbidity and mortality in individuals with DM. Macrovascular complications include myocardial infarction, peripheral vascular disease, stroke, and diabetic foot. Microvascular complications involve especially diabetic retinopathy, neuropathy, and nephropathy [5,6]. Accumulated evidence suggests that diabetic bone disease represents an important chronic microvascular complication of DM [7,8]. It is mainly manifested by an elevated risk of fractures caused by functional changes of bone cells and bone marrow adiposity, which affect several determinants of bone strength, microarchitecture, and geometry, together leading to bone deterioration [2,4,9,10]. As a result, individuals with both T1DM and T2DM have poor bone quality and health [10,11]. Individuals with T1DM generally possess low bone mineral density (BMD), although the relatively small decrease in BMD does not fully explain the increase in fracture risk. Conversely, subjects with T2DM have normal or even higher BMD compared with healthy individuals. These observations indicate that factors other than bone mass may affect fracture risk. These factors include poor glycemic control, disease duration, presence of diabetic complications, and some antidiabetic drugs [4].

Chronic hyperglycemia in DM enhances the generation of reactive oxygen species (ROS) by glucose autoxidation. Furthermore, ROS generation can be associated with the production of advanced glycation end products (AGEs), dysregulation of the polyol (sorbitol-aldose reductase) pathway, and elevated production of peroxidized lipids. Under conditions of oxidative stress, all intermediates of the polyol pathway can glycate proteins, leading to the formation of AGEs, which inhibit bone remodeling and indirectly suppress osteoblast activity and osteocyte apoptosis [8,12,13]. Osteocytes exposed to high concentrations of glucose elevate the expression of sclerostin, which inhibits bone formation via antagonizing the canonical Wnt/β catenin signaling pathway. Osteocyte vulnerability to AGEs raises sclerostin and reduces receptor activator of nuclear factor κB ligand (RANKL) expression, which is essential for osteoclast formation, differentiation, and survival [14]. Moreover, hyperglycemia weakens the response of osteocytes to mechanical stimulation, a mechanism crucial for their function [9].

Overall, the polyol pathway serves as a major link responsible for glucose toxicity [15,16]. Strong scientific evidence points to a primary role for aldose reductase (AR), the first enzyme of the polyol pathway, in the cascade of metabolic imbalances responsible for the harmful effects of hyperglycemia. Therefore, AR is considered an important therapeutic target [17]. Many AR inhibitors have been developed as potential therapeutics for several secondary diabetic complications [18,19,20,21]. 3-Mercapto-5H-1,2,4-triazino [5,6-b] indole-5-acetic acid (cemtirestat) was designed and patented as a highly selective and potent AR inhibitor with antioxidant activity [22,23]. The neuroprotective impact of cemtirestat was demonstrated in experimental rat models of DM [24]. According to Prnova et al. [25], prolonged cemtirestat treatment reduced the symptoms of peripheral neuropathy, inhibited sorbitol accumulation in erythrocytes, alleviated hypertriglyceridemia, and lowered indices of oxidative stress in streptozotocin (STZ)-induced diabetic rats. In Zucker diabetic fatty (ZDF) rats, treatment with cemtirestat partially inhibited the accumulation of sorbitol in erythrocytes and normalized symptoms of peripheral neuropathy [26]. The impact of cemtirestat supplementation on diabetic bone disease is still unknown.

Therefore, the aim of our study was to examine for the first time the effects of prolonged treatment with cemtirestat on bone quality in non-diabetic and diabetic rats. The streptozotocin (STZ)-induced rat model was used to simulate uncontrolled T1DM in our experiment.

## 2. Results

### 2.1. Blood Biochemistry

**Figure 1:**
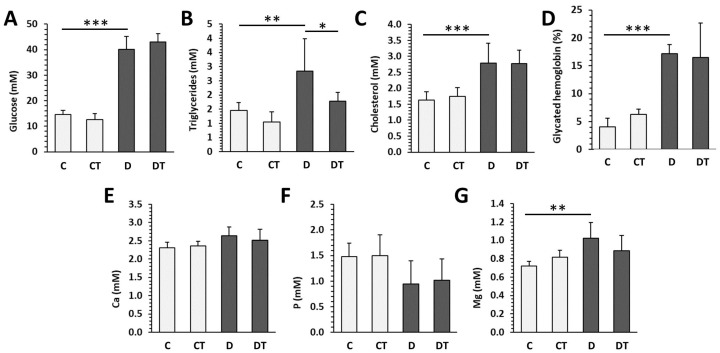
Biochemical parameters examined in non-diabetic rats (C), those treated with cemtirestat (CT), STZ-induced diabetic rats (D), and those treated with cemtirestat (DT). (**A**) Blood glucose, (**B**) triglycerides, (**C**) cholesterol, (**D**) glycated hemoglobin, (**E**) calcium, (**F**) phosphorus, and (**G**) magnesium. The values are represented as mean ± SD. Only differences between groups C and D, as well as C and CT, and D and DT are indicated (* *p* < 0.05, ** *p* < 0.01, *** *p* < 0.001).

Persistent hyperglycemia over 30 mmol/L was observed in both groups of diabetic rats throughout the whole experiment. Treatment of animals with cemtirestat did not significantly affect plasma glucose levels in either the non-diabetic or diabetic groups. Severe hyperglycemia was reflected by values of glycated hemoglobin over 17%. Significantly higher levels of plasma glucose, triglycerides, cholesterol, and Mg were reported in the D (diabetic untreated) group versus the C (non-diabetic untreated) group. The concentrations of Ca and P were not affected by T1DM. Cemtirestat treatment lowered plasma triglyceride levels in diabetic rats; other parameters were without significant changes. In non-diabetic rats, no effect of cemtirestat supplementation was noted (Figure 1).

### 2.2. Macroscopic and Densitometric Indicators

**Figure 2:**
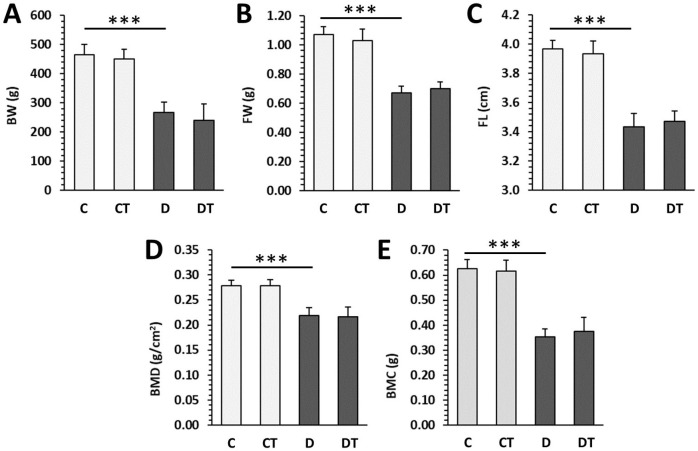
Macroscopic and densitometric parameters in non-diabetic rats (C), those treated with cemtirestat (CT), STZ-induced diabetic rats (D), and those treated with cemtirestat (DT). (**A**) Body weight (BW), (**B**) femoral weight (FW), (**C**) femoral length (FL), (**D**) bone mineral density (BMD), and (**E**) bone mineral content (BMC). The values are represented as mean ± SD. Only differences between groups C and D, as well as C and CT, and D and DT are indicated (*** *p* < 0.001).

Values for BW, FW, FL, BMD, and BMC were significantly reduced in the D group compared with the C group. No significant differences in parameters mentioned above were found between cemtirestat-treated and untreated groups (DT—diabetic treated versus D and CT—non-diabetic treated versus C), although cemtirestat supplementation resulted in a slight, but insignificant, improvement in BMC in diabetic rats. The results are illustrated in Figure 2.

### 2.3. Microcomputed Tomography

**Figure 3:**
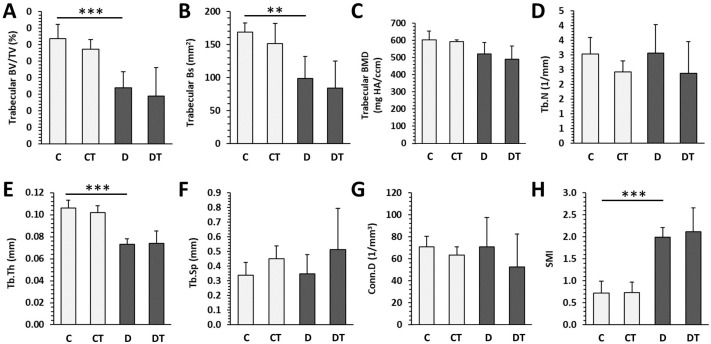
Micro-CT trabecular parameters in non-diabetic rats (C), those treated with cemtirestat (CT), STZ-induced diabetic rats (D), and those treated with cemtirestat (DT). (**A**) Volume fraction (bone volume/total volume; BV/TV), (**B**) bone surface (Bs), (**C**) bone mineral density (BMD), (**D**) trabecular number (Tb.N), (**E**) trabecular thickness (Tb.Th), (**F**) trabecular separation (Tb.Sp), (**G**) connectivity density (Conn.D), and (**H**) structure model index (SMI). The values are represented as mean ± SD. Only differences between groups C and D, as well as C and CT, and D and DT are indicated (** *p* < 0.01, *** *p* < 0.001).

**Figure 4:**
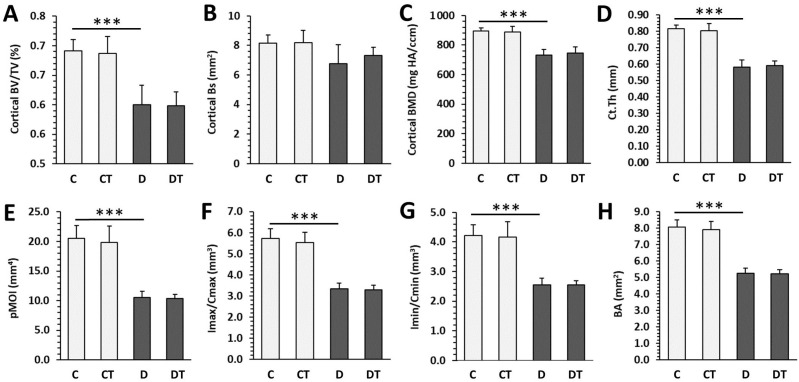
Micro-CT cortical parameters in non-diabetic rats (C), those treated with cemtirestat (CT), STZ-induced diabetic rats (D), and those treated with cemtirestat (DT). (**A**) Volume fraction (bone volume/total volume; BV/TV), (**B**) bone surface (Bs), (**C**) bone mineral density (BMD), (**D**) cortical bone thickness (Ct.Th), (**E**) polar moment of inertia (pMOI), (**F**) maximum loading resistance (Imax/Cmax), (**G**) minimum loading resistance (Imin/Cmin), and (**H**) bone area (BA). The values are represented as mean ± SD. Only differences between groups C and D, as well as C and CT, and D and DT are indicated (*** *p* < 0.001).

**Figure 5:**
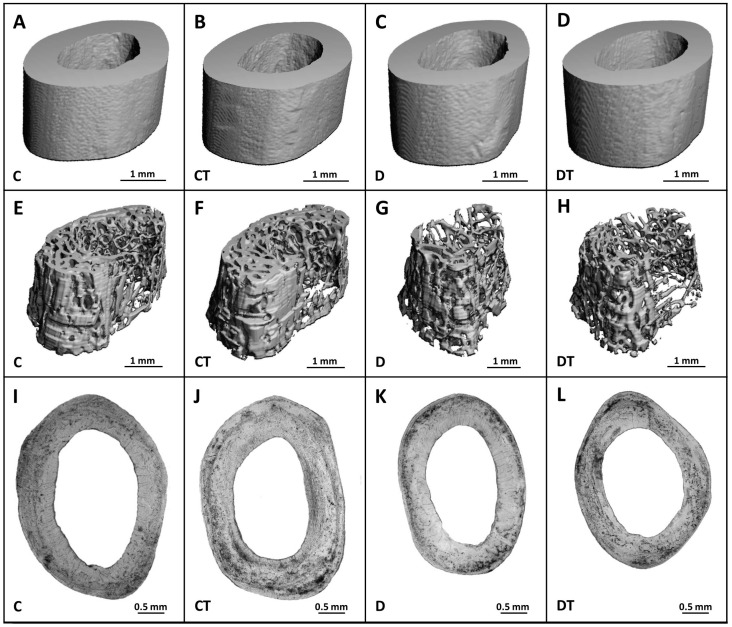
Micro-CT images of the cortical (**A**–**D**) and trabecular (**E**–**H**) bone and microscopic images of the cortical bone (**I**–**L**) in non-diabetic rats (C), those treated with cemtirestat (CT), STZ-induced diabetic rats (D), and those treated with cemtirestat (DT).

Significantly decreased trabecular BV/TV, Bs, Tb.Th and increased SMI were determined in rats from the D group versus the C group. T1DM had no effect on trabecular BMD, Tb.N, Tb.Sp, and Conn.D (Figure 3). Cemtirestat treatment did not significantly affect any of the measured parameters in trabecular bone in non-diabetic and diabetic rats. Significantly reduced cortical BV/TV, BMD, Ct.Th, pMOI, Imax/Cmax, Imin/Cmin, and BA were observed in the D group compared with the C group. Cortical Bs did not differ between rats from the D and C groups. Even in the case of cortical bone, cemtirestat supplementation did not have a significant effect on all investigated parameters in diabetic rats. The findings are documented in Figure 4. Representative reconstructed 3D images are shown in Figure 5.

### 2.4. Histomorphometry

**Figure 6:**
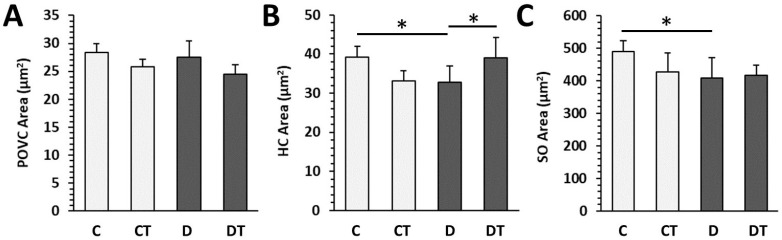
Histomorphometrical parameters in non-diabetic rats (C), those treated with cemtirestat (CT), STZ-induced diabetic rats (D), and those treated with cemtirestat (DT). (**A**) Primary osteons’ vascular canals (POVC) area, (**B**) Haversian canals (HC) area, and (**C**) secondary osteons (SO) area. The values are represented as mean ± SD. Only differences between groups C and D, as well as C and CT, and D and DT are indicated (* *p* < 0.05).

Histological structure of cortical bone did not differ between rats from the D and C groups. Both bone surfaces were predominantly composed of bone lamellae and osteocyte lacunae. A few secondary osteons (SO) and a higher number of primary osteons (PO) were most often identified in the central region. Rats from the D group had lower areas of HC and SO compared with the C group. Treatment with cemtirestat elevated the HC area in diabetic rats (Figure 6).

### 2.5. Bone Mechanical Properties

**Figure 7:**
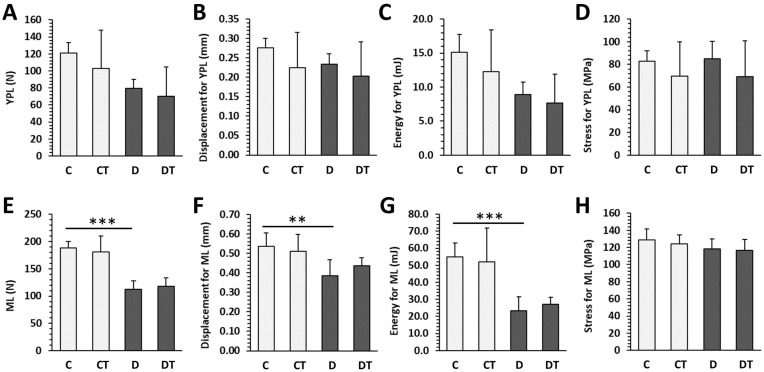
Mechanical properties of femoral bones in non-diabetic rats (C), those treated with cemtirestat (CT), STZ-induced diabetic rats (D), and those treated with cemtirestat (DT). (**A**–**D**) Yield point load (YPL) displacement, energy, and stress, (**E**–**H**) maximum load (ML) displacement, energy and, stress. The values are represented as mean ± SD. Only differences between groups C and D, as well as C and CT, and D and DT are indicated (** *p* < 0.01, *** *p* < 0.001).

Significant reductions in the values of maximum load, displacement, and energy for maximum load were noted in the D group versus the C group. Cemtirestat supplementation did not significantly affect bone mechanical properties in non-diabetic and diabetic rats (Figure 7).

## 3. Discussion

Rats with T1DM induced by pancreatic β cell-destroying drugs, including alloxan and STZ, belong to the most commonly used animal models [9]. Using an STZ-injected rat model, a beneficial effect of cemtirestat treatment on peripheral neuropathy, a serious microvascular diabetic complication, has already been described [25]. Because diabetic bone disease is also considered an important microvascular complication of DM, our study aimed to investigate the impacts of prolonged cemtirestat supplementation on bone parameters, reflecting bone quality and health in non-diabetic rats and rats with STZ-induced diabetes. First, we found that cemtirestat treatment did not affect all measured parameters in non-diabetic rats, indicating that this drug is safe.

In our study, higher levels of plasma glucose, triglycerides, cholesterol, glycated hemoglobin, and Mg were determined in diabetic versus non-diabetic rats. Equally, Prnova et al. [25] stated elevated levels of the aforementioned parameters in STZ-injected rats. Pournagi et al. [27] and Rivoira et al. [28] also reported higher blood glucose levels in STZ-induced diabetic rats versus controls. In patients with T1DM, increased plasma triglyceride concentrations have also been identified [29]. Consistent with the findings of Rivoira et al. [28], our study also found no significant changes in plasma Ca and P levels between diabetic and non-diabetic rats. Cemtirestat treatment only reduced levels of plasma triglycerides in diabetic rats, identical to the results of Prnova et al. [25]. Generally, significantly low lipoprotein lipase activities from adipose tissue were determined in STZ-induced diabetic rats [30]. According to Tsutsumi et al. [31], the lipoprotein lipase (LPL) activator NO-1886 raised post-heparin plasma LPL activity, resulting in a decrease in plasma triglycerides. High levels of plasma triglycerides are considered a risk factor for diabetic neuropathy. However, the association between hypertriglyceridemia and diabetic bone disease, such as that determined by BMD, remains controversial [32].

Consistent with our study, the findings of Pournagi et al. [27], Sha et al. [33], and Rivoira et al. [28] also showed reduced BW in STZ-injected rats compared with controls. Experimental T1DM is known to have a harmful impact on bone size [34]. In our study, FL and FW were significantly lower in diabetic rats. Lucas [35] also identified a shorter femur in STZ-induced diabetic rats. However, treatment with cemtirestat did not reverse this bone alteration in diabetic rats. 

It is widely accepted that T1DM induces a decrease in BMD and BMC [4,36], which was also confirmed in our study. Similarly, Rivoira et al. [28] and Londzin et al. [37] reported lower BMD and BMC values in rats with STZ-induced diabetes compared with controls. In any case, cemtirestat supplementation did not significantly improve BMD and BMC in diabetic rats, although a slight but non-significant improvement in BMC was observed. It is proven that adult T1DM patients suffering from microvascular complications had lower BMD than patients without microvascular disease [4,38], indicating a role of bone vascularization in the pathogenesis of diabetic bone disease. 

In rodent models of T1DM, deletion of the insulin receptor from osteoblasts leads to abnormal trabecular bone structure and raised bone fragility [39]. In patients with T1DM, trabecular bone quality was also lower compared with healthy subjects [40]. In our study, significantly decreased trabecular BV/TV, Bs, Tb.Th, cortical BV/TV, BMD, Ct.Th, pMOI, Imax/Cmax, Imin/Cmin, BA, and increased SMI values were recorded in diabetic versus non-diabetic rats. A strong deleterious effect of T1DM on the trabecular bone microstructure of rats was also identified by Londzin et al. [37], who noted significantly reduced values of BV/TV and Tb.Th. Similarly, Rivoira et al. [28] found decreased BV/TV, Tb.N, and Tb.Th and higher Tb.Sp values in a rat model of T1DM. Shanbhogue et al. [41] demonstrated not only lower Tb.Th, but also cortical BMD and Ct.Th in patients with T1DM, similar to our study. Cemtirestat treatment did not favorably affect all bone parameters mentioned above in STZ-induced diabetic rats.

Our results from the histological analysis of cortical bone are in agreement with other research in rodents [8,11,42,43,44]. We determined significantly decreased areas of HC and SO in diabetic versus non-diabetic rats. A lower HC area may indicate reduced blood supply. According to Nguyen and Barak [45], the size of the HC is determined by the size of the blood vessel(s) it carries, which have a minimum diameter limit, and thus smaller SO need relatively larger canals. Reduced SO size may be consistent with an elevated risk of fragility fractures [46] due to the accumulation of AGEs, altering collagen properties. According to Romero [47], vascular dysfunction is a major cause of morbidity and mortality in diabetic patients. The pathological process is characterized by disrupted production of the vasodilator and antiaggregation factor nitric oxide (NO) in endothelial cells and/or reduced bioavailability of NO. NO is the main regulator of vascular tone and integrity. Peng et al. [48] revealed reduced angiogenesis accompanied by a lower number of blood vessels in the femurs of STZ-induced diabetic mice. Jannapureddy et al. [49] established the central role of the aldose reductase pathway as a key mediator of endothelial dysfunction, disturbed endothelium-dependent relaxation, cell adhesion, and inflammatory events in diabetic blood vessels. The vascular structures in the HC show typical capillary features and are often paired. They are generally fenestrated, lined by a partial layer of endothelial cells, and bounded by a continuous thick basement membrane that limits ion transport through the capillary [50]. Overall, HC and SO are the results of the bone remodeling process. Generally, bone remodeling is closely related to vascular remodeling, which means that blood vessels in the HC are able to modify their structure [8]. In accordance with our results, cemtirestat treatment raised HC area in diabetic rats, indicating increased bone remodeling and a possible vasodilatory effect of cemtirestat on vascular structures in HC. Aldose reductase inhibitors (ARIs) have been shown to possess some beneficial effects on blood vessels, particularly in the eyes and kidneys, but there is limited evidence to suggest that they have a direct effect on blood vessels in bone. 

We found significant reductions in the values of maximum load, and displacement and energy for maximum load in the femoral diaphysis in diabetic versus non-diabetic rats. Londzin et al. [37] also stated an adverse impact of T1DM on the mechanical properties of the femoral diaphysis, finding a reduction in the values of load and energy for the yield point in STZ-injected rats. In our study, cemtirestat supplementation did not significantly affect bone mechanical properties in diabetic rats. 

Recently, a number of ARIs have been developed as potential candidates for the treatment of several diabetic complications. It has been suggested that they may have indirect impacts on bone metabolism by reducing oxidative stress and inflammation. So far, the only ARI on the market is epalrestat, which is used to treat patients with diabetic neuropathy and is only sold in Eastern countries [51,52]. According to Inaba et al. [53], epalrestat has been shown to reduce the temporary increase in bone resorption markers and subsequently elevated bone volume in a rat model of DM. Glajchen et al. [54] evaluated the impacts of another ARI sorbinil on biochemical and bone histomorphometric parameters in STZ-induced diabetic rats. Their findings indicate that sorbinil did not affect all examined parameters, and the lack of its beneficial effect would suggest that the polyol pathway may not be a significant factor contributing to the pathogenesis of diabetic bone disease. The aforementioned studies indicate that ARIs may have only low potential as therapeutics for diabetic bone disease, but further research is needed to fully understand their effects on bone quality and health in DM.

## 4. Materials and Methods

### 4.1. Animals and Experimental Procedures

Male Wistar rats (1.5 months old) were bred at the Slovak Academy of Sciences, Department of Toxicology and Laboratory Animal Breeding, Dobrá Voda. Rats were housed in T4 Velaz cages (Prague, Czech Republic) in groups of two. Temperature (22–24 °C), photoperiod (12/12 h light/dark cycle), and relative humidity (40–70%) were regulated and monitored. Standard rodent chow and tap water were provided ad libitum. Experimental diabetes was induced by triple i.p. doses of STZ (30 mg/kg, Sigma-Aldrich, St. Louis, MO, USA) for three days. STZ was dissolved in citrate buffer (pH 4.5, 0.1 M). Citrate buffer was administered to control rats as well. All animals with plasma glucose levels >15 mM were considered diabetic (three days after the last dose of STZ) and were included in the study.

Control and diabetic rats were divided into four groups: untreated non-diabetic rats (C group, n = 6), non-diabetic rats treated with cemtirestat (Apollo Scientific Ltd., Bredbury, UK) dissolved in drinking water (CT group, n = 6, 6.4 mg/kg/day), untreated diabetic rats (D group, n = 6), and diabetic rats treated with cemtirestat (DT group, n = six, 6.8 mg/kg/day) as described by Prnova et al. [25]. Treatment lasted 120 days.

### 4.2. Blood Biochemistry

Blood was taken by cardiac puncture into a heparinized tube (8 mL), immediately put on ice and centrifuged at 4 °C, 15 min, 1500 rpm. After centrifugation, the plasma was collected and frozen at −80 °C until the analysis. Plasma glucose level was determined using a Glucose GOD 1500 enzymatic colorimetric assay kit (PLIVA-Lachema Diagnostika, Brno, Czech Republic). Glycated hemoglobin was determined by using a rat HbA1c kit of Crystal Chem Inc (Elk Grove Village, IL, USA). Plasma triglycerides, cholesterol, calcium (Ca), phosphorus (P), and magnesium (Mg) levels were assayed by Laboratoria s.r.o., Piestany, Slovakia. 

### 4.3. Macroscopic and Densitometric Indicators

At the end of the experiment, body weights (BW) of rats from all groups were recorded. Both femoral bones (n = 48) were used to determine macroscopic (femoral weight—FW, femoral length—FL) and densitometric (bone mineral density—BMD, bone mineral content—BMC) indicators. Dual energy X-ray absorptiometry (DXA) using a Hologic QDR^®^-4500 was used to measure BMD and BMC.

### 4.4. Microcomputed Tomography 

Microcomputed tomography (μCT 50, Scanco Medical, Brüttisellen, Switzerland) was applied to evaluate the microstructure of both cortical and trabecular bone tissues. Before analysis, femoral bones were wrapped in gauze soaked in PBS solution and stored below −18 °C. High resolution scans with a voxel size of 14.8 μm were acquired. Because of significantly lower FL in diabetic rats, the region of interest for cortical bone analysis was selected at a distance of 10.8 mm from the end of the growth plate (EGP), whereas that for non-diabetic rats was selected at a distance of 12.4 mm from the EGP and extending 2 mm at the femoral midshaft. Trabecular bone was scanned at a distance of 2.5 mm from the EGP for diabetic rats, whereas that for non-diabetic ones was scanned at a distance of 2.9 mm from the EGP and extending 2 mm. Scanning parameters included 70 kVp voltage, 200 mA current, 300 ms integration time, 0.5 mm aluminum filter. Quantitative analysis was performed using Scanco microCT Evaluation Program V6.6. Trabecular bone mass measurements involved bone volume fraction (BV/TV), bone surface (Bs), trabecular bone mineral density (BMD), trabecular number (Tb.N), thickness (Tb.Th), and separation (Tb.Sp), whereas those of trabecular microarchitecture incorporated connectivity density (Conn.D) and structure model index (SMI). Measurements of cortical microarchitecture and geometry included bone volume fraction (BV/TV), bone surface (Bs), cortical BMD, cortical bone thickness (Ct.Th), bone area (BA), polar moment of inertia (pMOI), and maximum (Imax/Cmax) and minimum (Imin/Cmin) loading resistance. 

### 4.5. Histomorphometry

For histological analysis of the cortical bone, right femoral bones (n = 24) were cut at the diaphyseal midshaft. Transverse thin sections (70–80 μm) were prepared and visualized under light microscope (Leica DM 2000, Wetzlar, Germany) as previously described [55]. The quantitative 2D parameters (areas of primary osteons’ vascular canals—POVC, Haversian canals—HC, and intact secondary osteons—SO) were assessed using software Motic Images Plus 2.0 ML (Motic China Group Co., Ltd., Xiamen, China). 

### 4.6. Bone Mechanical Properties

A three-point bending test was used to evaluate the mechanical properties of the left femoral diaphysis using an Instron 3342 500 N apparatus (Instron, Norwood, MA, USA). Bluehill 2 version 2.14 software (Instron, Norwood, MA, USA) was used for data analysis. The femur was placed on two supporting points (the distance between those points was 16 mm). The load was applied perpendicularly to the long axis of the bone in the middle of the femoral length. After preconditioning to obtain steady positioning of the bone, a proper test started, with a displacement rate of 0.01 mm/s and a sampling rate of 100 Hz. The following extrinsic (depending on bone size) parameters were assessed: the load, displacement and energy for the yield point (0.05% offset), and maximum load point. The stress for the yield point and the stress for the maximum load point (intrinsic parameters, independent of bone size) were also determined. To evaluate the moment of inertia in the break-section, necessary for the stress calculations, it was assumed that the femoral diaphysis was a circular beam, and the mean diameter of the femoral diaphysis in the bone mid-length was measured with the use of a digital caliper (Topex, Warsaw, Poland).

### 4.7. Statistical Analysis

SPSS Statistics 26.0 software (IBM, New York, NY, USA) was used to perform statistical analyses. Data were presented as mean ± standard deviation (SD). ANOVA with Tukey’s post hoc test was used to determine differences in all parameters examined. A P value of less than 0.05 (*), 0.01 (**), and 0.001 (***) was considered statistically significant.

## 5. Conclusions

Cemtirestat supplementation did not affect all bone parameters reflecting bone quality (biochemical, macroscopic, densitometric, micro-CT trabecular and cortical, histomorphometrical, those of mechanical properties) in non-diabetic rats. In STZ-induced diabetic rats, cemtirestat treatment significantly lowered only plasma triglycerides level and elevated the Haversian canal area. A slightly, but insignificantly, improved BMC was also identified. It can be concluded that the insufficient effect of cemtirestat treatment on bone quality in T1DM does not support its use in the therapy of this diabetic complication.

## Data Availability

Data is contained within the article.
